# Validation of a Thai artificial chatmate designed for cheering up the elderly during the COVID-19 pandemic

**DOI:** 10.12688/f1000research.127431.2

**Published:** 2024-01-11

**Authors:** Somrudee Deepaisarn, Ek-uma Imkome, Konlakorn Wongpatikaseree, Sumeth Yuenyong, Ploi Lakanavisid, Rangsiman Soonthornchaiva, Panida Yomaboot, Angkoon Angkoonsawaengsuk, Napawan Munpansa

**Affiliations:** 1 Thammasat University, Klong-luang, Pratumtane, 12120, Thailand; 2 Department of mental health and psychiatric nursing, Faculty of Nursing, Thammasat University, Klong-luang, Pratuntane, 12120, Thailand; 3 Department of Computer Engineering, Mahidol University, Salaya, Nakhon Pathom, 73170, Thailand; 4 Faculty of Medicine, Burapha University, Chon Buri, Chon Buri, 20131, Thailand; 5 Department of Psychiatry, Faculty of Medicine Siriraj Hospital, Mahidol University, Bangkok Noi, Bangkok, 10700, Thailand

**Keywords:** artificial chatmate, Elderly, mental health, COVID-19, health promotion

## Abstract

**Background:**

The COVID-19 pandemic severely affected populations of all age groups. The elderly are a high-risk group and are highly vulnerable to COVID-19. Assistive software chatbots can enhance the mental health status of the elderly by providing support and companionship. The objective of this study was to validate a Thai artificial chatmate for the elderly during the COVID-19 pandemic and floods.

**Methods:**

Chatbot design includes the establishment of a dataset and emotional word vectors in which data consisting of emotional sentences were converted into the word vector form using a pre-trained word2vec model. A word vector was then input into a convolutional neural network (CNN) and trained until the model converges using sentence embedding and similarity word segmentation. Sentence vectors were generated by averaging each word vector using an averaged vector method. For approximate similarity matching, the Annoy library was used to create the indices in tree sorting. Data were collected from 22 elderly and assessed by the Post-Study System Usability Questionnaire (PSSUQ).

**Results:**

The study revealed that 72.73% of the respondents found the chatbot easy to learn and use, 63.64% of the respondents found the chatbot can autonomously determine the next course of action, and 59.09% of the respondents believed that troubleshooting guidelines were provided for overcoming errors. The accuracy of the chatbot providing a reasonable response is 56.20±13.99%.

**Conclusions:**

Most users were satisfied with the chatbot system. The proposed chatbot provided considerable essential insights into the development of assistance systems for the elderly during the coronavirus pandemic (COVID-19) and during the period of national disasters. The model can be expanded to other applications in the future.

## Introduction

The COVID-19 pandemic has severely affected global public health, economics, education, and society. During the pandemic, technology’s use for companionship attracted considerable research attention. In addition to physical healthcare, ensuring the population’s mental health during the pandemic is critical. Therefore, this research developed personalized healthcare systems to support the elderly in their daily tasks and promote active and healthy aging. Validity, reliability, and sustainable measures are essential for the healthy aging of the population ( Martinho *et al.*, 2022).

As a result of the impact of COVID-19 outbreaks in Thailand since early 2020, the Ministry of Higher Education, Science, Research and Innovation promoted the health status and economic underpinnings of national strategic projects by using integrated development schemes related to four elements: a) creation of health technologies and applications for promoting community health, b) promotion of goods in community, c) promotion of tourism and d) promotion of circular economy. For this strategy, Thammasat University has applied the technology, knowledge, and expertise to help people in the healthcare domain. Every risk is assessed considering possible effects so that the risk can be addressed appropriately. The pilot project reveals that an efficient health technology system should be developed to deduce strategies to engage, persuade, and motivate older adults to promote positive behavioral changes and increase their awareness of other health conditions. The state quarantine and the physical distancing regulations of the first wave of the pandemic in Thailand significantly impact the elderly’s mental health status. These issues need to be addressed. Considering the Thai culture, older adults with problematic health conditions are typically assumed to spend most of their time alone in a private household, resulting in loneliness. Such people depend on other family members and require personal assistance to complete daily tasks and general chores. Thus, technological influence is essential in improving the preventive measures against mental health problems in the elderly population.

Artificial Intelligence (AI) has been incorporated into many sciences and arts fields, including healthcare and support systems ( Kusal *et al.*, 2022). Furthermore, AI technologies have been used in hospitals as well as health and well-being sectors for mental health screening ( Otero-González, Pacheco-Lorenzo, Fernández-Iglesias, & Anido-Rifón, 2024). The implementation of AI in healthcare can provide cost-effective solutions for improving the health of people in modern society ( Väänänen, Haataja, Vehviläinen-Julkunen and Toivanen, 2021). A chatbot can interact with the elderly and clarify their needs and emotions across the database and analytic system. Chatbots can replace the workforce required in many sectors, including chat. Chatbot exhibits considerable potential for integration into clinical practice and healthcare services. In medical and health research, chatbots have been used for food suggestions ( Fadhil, 2018), smoking cessation ( Wang *et al.*, 2018), and cognitive behavior therapy ( Fitzpatrick *et al.*, 2017). Similar approaches have supported focused groups with health problems ( Kumar and Rosé, 2014). Chatbot assists young adult or teenager groups in answering socially sensitive questions related to sexual health, drugs, and alcohol consumption. Users can “talk” to a programmed chatbot without fewer concerns and anxiety about sharing personal information with others ( Crutzen *et al.*, 2011). A well-designed chatbot can also give accurate guidance to the users. Thus, the first barrier to be overcome is the user's trust in the system. This can only be achieved if the system can ensure that the private information is non-identifiable and non-disclosable by any other means apart from undergoing a screening procedure that benefits the user and is authorized by that user only.

Furthermore, chatbot systems provide a considerable advantage to people who want to obtain health information compared with conventional search engines ( Bickmore *et al.*, 2016) for enhancing their understanding of their health conditions, improving health-related behaviors, and making decisions for treatments.

Chatbot applications in primary healthcare are more cost-effective than other solutions, such as medical nursing and medical robots. Many people/patients can access such tools using various chatbot platforms, including text and voice interfaces ( Kreps and Neuhauser, 2010). Voice interfaces are more efficient, especially for mental health counseling ( Wang *et al.*, 2015). Such systems provide suggestions for improving users’ health and quality of life. Therefore, in this study, “Ai-aun”, the artificial chatmate, was developed to promote the health of elderly people, especially during the COVID-19 pandemic and floods. The chatbots assess the similarity of the content cited in a spoken sentence and identified as an essential part of speech. Ai-aun can help the elderly adapt to stressful situations during the COVID-19 pandemic by maintaining good mental health, promoting healthy routines, and preventing dementia. The objective of this study was to validate the Thai artificial chatmate, “Ai-aun”, for the elderly people during the COVID-19 pandemic and during common disasters, such as floods. However, the use of the chatmate is not limited to such emergencies.

## Methods

### Chatbot architecture

In this study, a natural interface was used. The focus was on developing computers to understand human nature. Originally developed for linguistics, psychology, and computer science, natural interfaces can be applied to various fields where humans can conveniently communicate with computers/machines and convey empathy. Virtual reality is creating a virtual image or a simulation of an event using a computer system based on the interpretation of an ordinary human language. In this work, the development of the voice interface system allows humans to speak or operate efficiently with mobile devices.

Chatbot mechanism can respond to and aid users in comprehending the level of their health status and assist the users with the decision-making process to take care of themselves and improve their health. In such systems, user voice data are the inputs ( *i.e.*, voice interface). Next, the input is translated into text information. The association between the input text and the database is mapped using a trained model. This model is used to produce the output in the form of text. This result is translated back into voice and promptly conveyed to the user. The process is repeated for all user-triggered inputs, and each triggered input is regarded as an independent conversation.

**Figure 1.**
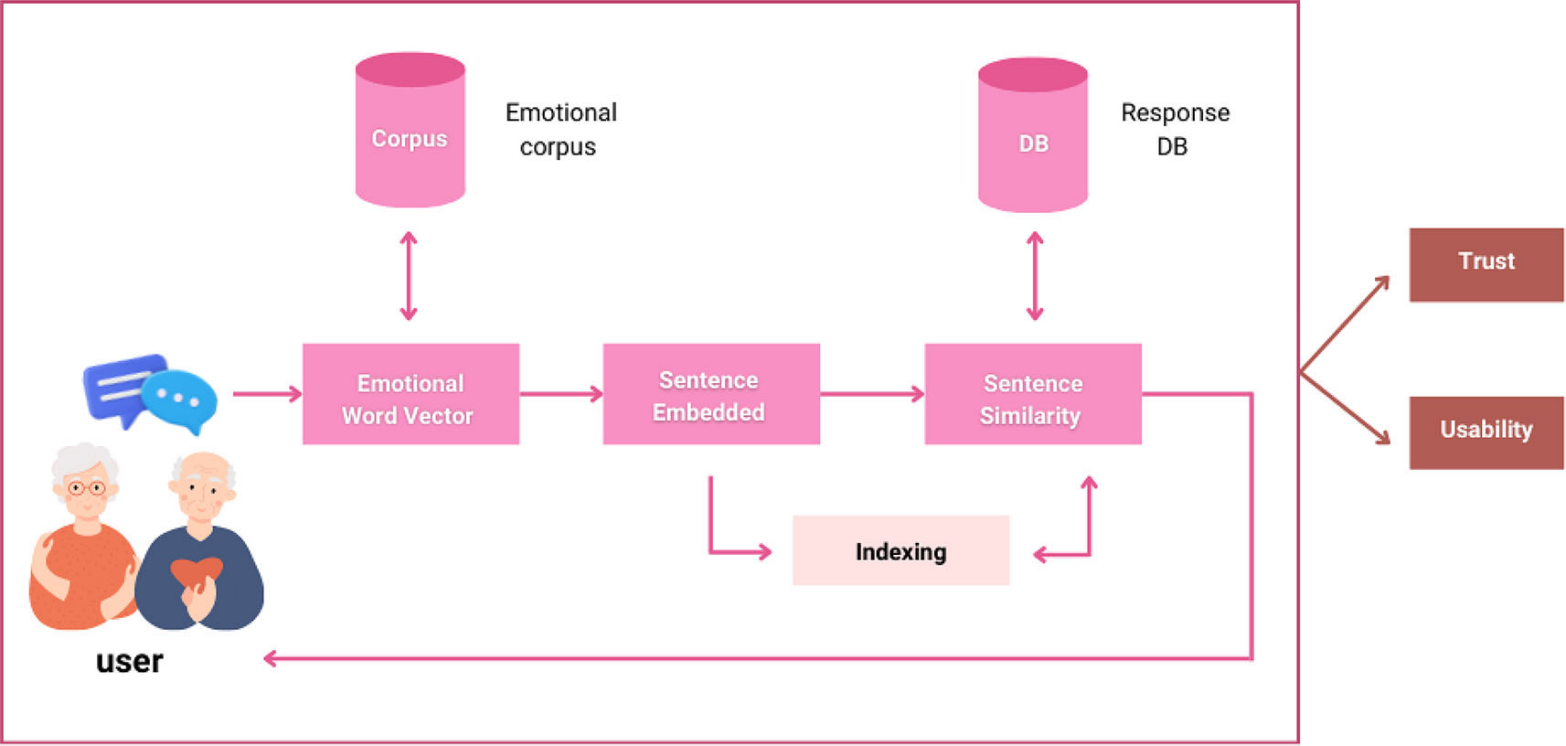
Conceptual framework of the chatbot.

The Ai-aun chatbot was developed using natural language processing, virtual reality, and hybrid AI systems. Trust in the system, and its usability is the central cores of this chatbot. In this study, a novel framework was created for the chatbot to produce a reasonable response to a user's message regarding content and emotional characteristics. To build the Ai-aun chatbot, three consecutive modules: namely emotional word vector, sentence embedding and similarity, and approximate similarity matching, were adopted. Figure 1 shows the framework with all components of the chatbot. The elderly can take care of themselves in six general areas: general health, medication, diet, exercising, sleep and rest, and family relationship. The chatbot can provide timely reminders to the elderly to take their medicine, exercise, and sleep to ensure they stay in good health, especially during the COVID-19 pandemic.

### Algorithms and system design

The chatbot responds to the elderly user’s conversation to improve their mental health. The database system records and monitors all input information so that the authorized administrators can access it. The system can generate a report for administrators for analysis. After using the system, users are asked to complete a trust survey containing 15 questions to understand their trust, confidence, and the quality of conversation. The system performance was assessed using Qualtrics ( Corritore *et al.*, 2005). An effective interface should provide simple and easy-to-use features, well-structured, and robust outputs. Higher scores indicate higher trust and usability levels. This is the conventional way of validating a chatbot. A statistical technique was also applied to assess the proposed artificial chatmate reliably. Ai-aun comprises three modules: emotional word vector, sentence embedding and similarity, and approximate similarity matching.

*Module 1: Emotional word vector*

To convert a word into a vector for further analysis, data pre-processing and word2vec training are required.

The data pre-training was obtained from the 30 elderly volunteers on the U2T project. The research teams checked for accuracy (general health; 193 data sets; family; 115 datasets; medication; 122 datasets; diet; 137 datasets; exercise; 118 datasets; sleep and rest and other activities; 151 datasets). After obtaining the accurate data set, the researcher teams submitted data to the three experts for the process of content validity testing. The dataset was addressed as the expert comment for the training sessions.

In the pre-processing steps, raw input data are prepared. That is, text messages are proposed into a suitable format for analysis. These steps include word segmentation and stop word removal because the sentences were appropriate for content validity by three experts and the research teams before launching the language in NLP. Additionally, we need to check and remove some sentence errors during text pre-processing. This process was adjusted to suit the structure of the Thai language. Note that the variation of the user’s pronunciation can result in misinterpretation by the model and cause unintended outputs ( *i.e.,* amiss expression of emotion). This can be prevented with a proper pre-processing of data.

• In *Word segmentation,* texts that appear in the sentence are divided into individual words. Binary classification is applied to search for a unique character as the starting point of each word in an input sentence. Cutkurn was selected as the Thai word tokenization library for constructing this system. Cutkurn is based on a recurrent neural network (RNN) model.
• *Stop word removal* eliminates the words of minor significance in the sentence so that these words do not affect the sentence’s overall meaning. Examples of Thai-specific words in this category include the prefix or suffix of phrases expressing politeness or emphasis, conjunctions, and some ignorable adverbs.

Next, the word2vec functions allow an appropriate model to be trained from the raw input by trying to map the vector representations of words using the process of word embeddings ( Mikolov *et al.*, 2013). Skip-gram and Common Bag of Words (CBOW) are used. Skip-gram considers the meaning of a word to predict multiple consecutive words and vice versa for the CBOW technique.

The visual information in conversational agents may take affective states and thus is an appropriate indication of emojis in texts. The objective is to use emojis in the study for better interpretability of emotional user states, especially for short polysemous phrases of both positive and negative emotions in many scenarios. Understanding willingness, mood, and affect will help conventional agents respond to reliability, validity, and appropriateness. Additionally, Emojis are users’ reported labels of mood and affect to convey emotions in the context of communication interactions.

The working process of the emotional word vector module consists of two main steps.

• Step 1: In this step, emotional information is inserted into the pre-trained word vector to add the semantic meaning of the emotion into the vector of individual words, typically pre-trained on a large, standard data corpus. Therefore, the syntax and syntactic aspects of each word are accurately captured, for example, in a way that the vector of the word “dog” is far away from the vector of the word “doctor”. However, the emotional dimension is not captured satisfactorily. Users find that the vectors for the words “sad” and “happy” are closer because these are adjectives. Thus, if the user applies this pre-trained vector to the emotional chatbot, the results will be inaccurate in terms of emotion.
• Step 2: Word vectors from the trained word embedded models were re-adjusted via a Convolutional Neural Network (CNN) to improve the emotional dimension that should be optimized for each word. The first layer and word embedded layer constitute the first module. The weight of the word embedded layer is continuously optimized until converged in order to determine the emotional dimension.

*Module 2: Sentence embedding and similarity*

**Figure 2.**
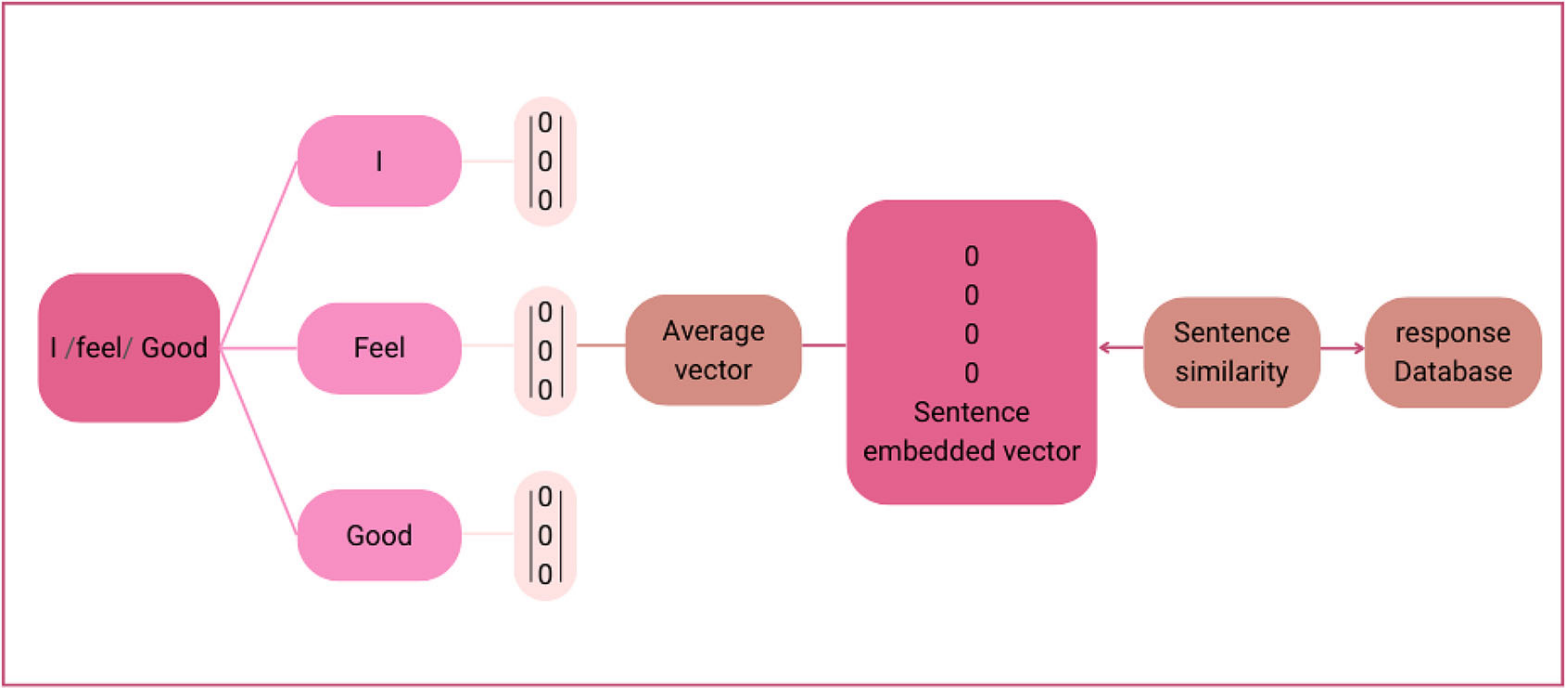
Sentence embedding and similarity.

The vector obtained from Module 1 is at the word level, which is converted into a vector of sentence embedding and similarity, as illustrated in Figure 2.

Words from the sentence were segmented using the Python library developed for Thai word segmentation, called “Cutkum”, which was developed by Treeratpituk (2017). Next, each word is converted into a word vector using the emotional word vector model created in Module 1. Then, a sentence vector is generated from averaged word vectors. Finally, the vector similarity between the input sentence and all the sentences in the response database is computed using cosine similarity according to Equation (1), where A and B are the sentence embedding vectors to be compared.

$$\text{Similarity}=\text{cosθ}=\frac{A∙B}{\left|A\right|\left|B\right|}$$ (1)

*Module 3: Approximate similarity matching*

Similarity matching consists of generating an index followed by the sorted-tree search. A sentence vector is compared to the existing library. Hence, an accurate answer can be suggested. However, real-time response is required for a chatbot to be sufficiently fast. This research uses the approximate nearest neighbors oh yeah (Annoy) library, where the vectors are rearranged in the response according to the index of the database. This exhibits a fast similar-items retrieval data structure that is ideal for use with chatbots with numerous sentences.

### Tool validation

*Ethical approval*

Ethical approval for the study was obtained from the Ethics Review Committee for Research Involving Human Research Participants, Thammasat University, Thailand (COA No. 115/2564) on November 4, 2021. The scope, risks, and benefits of the study were explained to all participants. Before data collection, written and verbal consent was obtained directly from participants. Participation was voluntary, and participant anonymity and confidentiality were ensured.

*Data collection*

The elderly people were selected as participants through purposive sampling on November 11, 2021. People aged above 60 years who speak Thai as their first language and can read Thai were selected. However, the ability to read Thai was a soft requirement because this study focused on communication through the voice interface. Those who were unable to participate in at least 80% of the experiment and had health problems that hindered participation in the experiment were excluded.

Twenty-two elderly participants, 18 women, and 4 men were selected from the population in Bang Krabue, Sam Khok, Pathum Thani, Thailand. The participants were interviewed to collect the sentences they would use to chat with the Ai-aun chatbot. Data collection was finalized with the saturation of the data. After that, the dataset was evaluated by the researchers (including computer scientists, physicians, psychologists, and psychiatric nurses) and submitted to three validators ( *i.e.,* a psychiatrist, a nurse who worked in the aging field, and a nursing lecturer in the aging field) to examine the content validity index. To qualify the datasets for the emotional word vector, sentence embedding and similarity word segmentation were performed to generate sentence vectors, followed by approximate similarity matching. Subsequently, the time spent with the chatbot, the rate of questions per minute, and the usability of the chatbot by the elderly were tested after 15 days of using the Ai-aun chatbot on a mobile device.

### Data analysis

• Demographic data were recorded, including age, gender, marital status, education, career, income, personal illness, social networking sites, and time on social networks.
• A Post-Study System Usability Questionnaire (PSSUQ) was used to measure the usability variable, which consists of 15 questionnaires to assess users’ satisfaction with the chatbot interface in terms of language, graphics, and simplicity of the user interface ( Lewis, 1995). Typically, PSSUQ scores range from 1 (strongly agree) to 7 (strongly disagree). The higher value means more excellent usability. The Content Validity Index (CVI) is 0.86.
• Training dataset for the conversational chatbot agent: A sample scheme of questions and responses was utilized for the dataset validation. An appropriate answer was selected from the volunteering elderly from the community for training and testing the chatbot model. The CVI for the dataset is 0.95.
• The time spent with the chatbot, recorded in the time log, was analyzed concerning the rate of questions per minute.

All parameters mentioned above were reported using descriptive statistics.

*Technical performance test for accuracy*

The accuracy of the chatbot providing reasonable responses was systematically tested by ten trained technicians. Each individual spoke 100 random questions based on how they would talk generally. The elderly conversations and answers from the chatbot were collected. One out of the four emotional states, *i.e.,* positive, neutral, frustrated, and negative, was determined for each sentence. The predicted scores for such emotional states were calculated.

## Results

The sample consisted of 22 elderly people. The mean age was 68.86±2.56 years old. Out of the total participants, 81.82% were women, and 18.18% were men. People with physical illnesses accounted for 72.73% of the samples. Diabetes mellitus is the most common chronic illness (45.45%) among the participants.

The time spent with the Ai-aun chatbot was approximately 0.20-0.30 s, and the rate of the user asking questions to Ai-aun was 0.28 questions per minute. The Ai-aun chatbot had a moderate usability level (see the summary Table 1). After 15 days of using the Ai-aun chatbot, 59.09% of the participants were delighted with the system, indicating that it had all the functionalities and capabilities they expected. Additionally, the accuracy of the chatbot in terms of providing a reasonable response was 56.20±13.99%. The predicting scores for the emotional states ranged between 0.50 and 1.00, with the mean predicted score of 0.79±0.17.

**Table 1.** Variables, measures, and the results of the study.

Variable	Measure	Result
1. The time spent with the chatbot	Time log	∼0.20-0.30 s
2. Rate of questions per minute	Number of questions/times taken	0.28 min ^-1^
3. Usability	PSSUQ	Moderate usability

The results revealed that 72.73% of the participants found the chatbot easy to learn and use, and 63.64% of participants thought the chatbot was sufficiently autonomous in determining its next course of action. In case of a system error, the system also provided troubleshooting guidelines according to 59.09% of participants.

## Discussion

In this study, the Ai-aun chatbot was developed to cheer up elderly people during the COVID-19 pandemic. The time spent with the chatbot, the rate of questions per minute, and the usability measured using the PSSUQ were the variables used in this study. The time spent was 0.2-0.3 s. The average rate of questions was 0.28 min ^-1^. The dataset for the Ai-aun chatbot was designed for short spoken sentences as the input, and the average rate of questions per minute was fast. The usability was at a moderate level. The participants were happy that the Ai-aun system had all the functionalities and capabilities they expected from a chatbot, and the application device was easy to use. Furthermore, a young girl’s voice was adopted along with a friendly, easy-to-use interface ( U2T, 2022). Therefore, familiarizing participants with the Ai-aun chatbot was essential. Elderly people were satisfied with the Ai-aun chatbot ( Mehta *et al.*, 2022). Moreover, a chatbot provides smooth conversation, including greeting, salutation, compromising, support, and guidance. For example, a simple sentence that sounds relaxing is added, such as “Have a nice day”, “Don’t worry…I will always be with you”, and “I love talking to you”. These sentences encouraged elderly people to interact with the Ai-aun. In addition, Ai-aun provides a feature for medication, rest, exercises, diet schedule, and reminders. The system was consistent with the mobile application developed by Puig (2021), +Approp, to support older and HIV-infected patients. It provides clinical history ( *e.g.,* vaccines and medication taken), educational feedback, and reminders of medical appointments. Positive responses were obtained regarding the usability and satisfaction of mobile healthcare applications.

**Figure 3.**
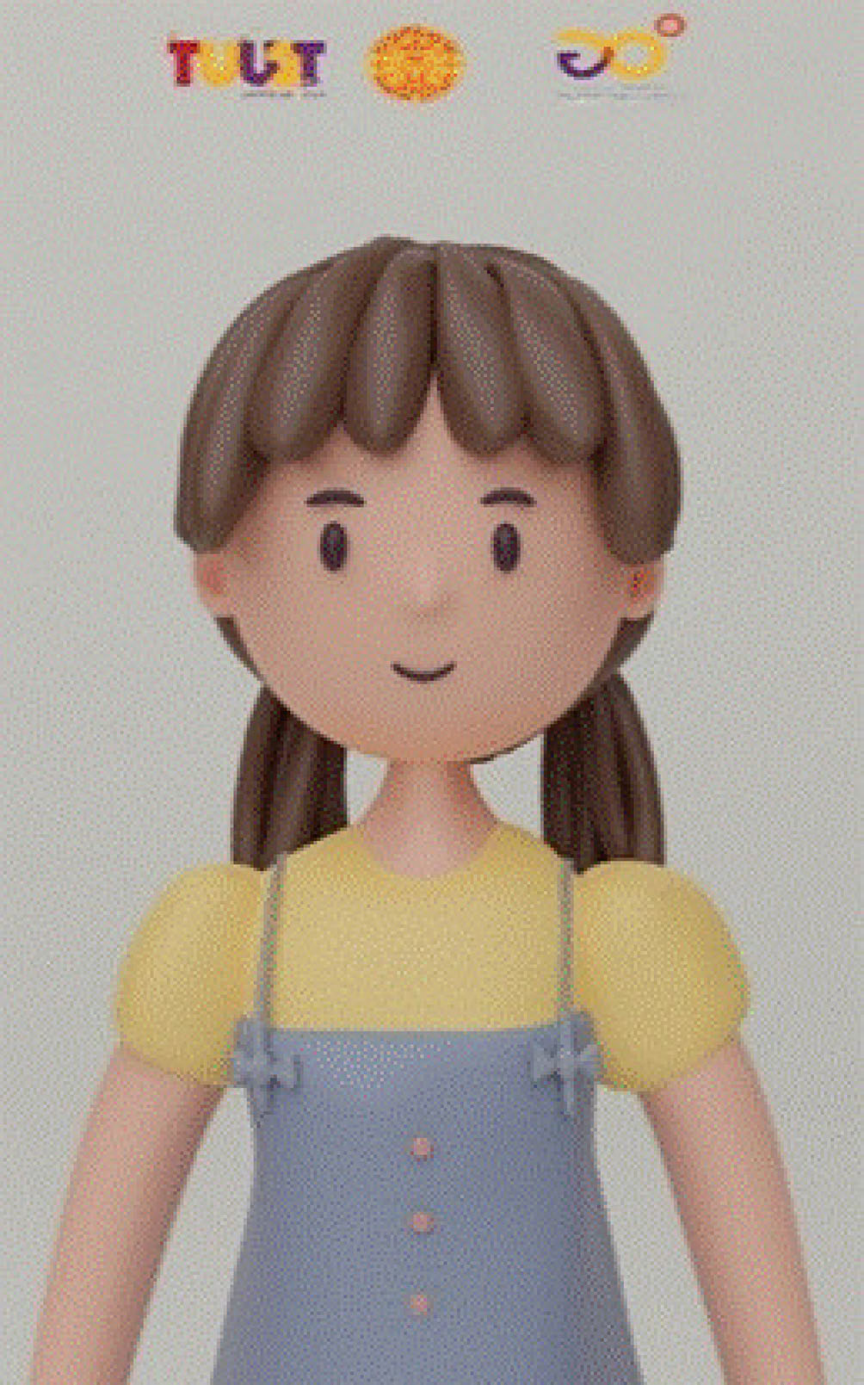
Ai-aun ( U2T, 2022). The application can be downloaded from the Google Play Store.

The Ai-aun chatbot was developed by a paradigm suitable for accepting natural language input and processing computer instructions to enable users to achieve their health goals ( Abdul-Kader and Woods, 2015; Fadhil, 2018; Kusal *et al.*, 2022). Participants enjoyed chatting with Ai-aun ( U2T, 2022) ( Figure 3). A positive experience and familiarity with the mobile application result in the satisfaction of use.

This study proposed a mobile chatbot system to support elderly people, which encourages the elderly to access and get familiarized with the technology to support their physical and mental health. The agent provides proactive functions, sending messages to help elderly users educate and relax in general health, taking medication, diet, family relationships, exercise, rest, and sleep. Reminiscence strategies are considered challenging when related to design interfaces, conversation logic, and meaningful metrics directed at elderly adults, which benefit from screening and educating them ( Kusal *et al.*, 2022; Moreno, Sánchez-Anguix, Alberola, Julián, & Botti, 2024; Otero-González, Pacheco-Lorenzo, Fernández-Iglesias, & Anido-Rifón, 2024). Having enhanced the technological experience of the elderly, they can interact directly with Ai-aun confidently and successfully receive responses related to promoting their health conditions. Four emotional states of the conversation were determined to reflect the user’s actual sentiment. The effectiveness of the response from the chatbot system in terms of progress, interactions, participation, and satisfaction was assessed. Therefore, the system can be expanded to incorporate more functions that align with the users’ needs and applied to more participants.

For the current configuration, three consecutive modules: emotional word vector, sentence embedding and similarity word segmentation, and approximate similarity matching, were used to elicit a response from the database ( Ng, Chia, Yap, & Goh, 2022). The accuracy of the chatbot providing a reasonable response was 56.20±13.99% which depends on the dialogue between the individual and chatbot. Such accuracy is typical for a chatbot that is expected to handle various sentences and sentiments. The accuracy can be improved by increasing the training dataset size to cover a broad variety of conversations. Based on this study, Ai-aun’s model – of the elderly language phenomenon should be studied further. The elderly’s visual and auditory impairment may be an obstacle to the kind of chatbot models.

## Conclusion

This study revealed a moderate usability level. Several users were satisfied with the chatbot system, which provided basic information. They can be used as guidance in developing care and assistance systems for the elderly in the community during the coronavirus pandemic (COVID-19) and in national disasters, such as flood situations. Therefore, the chatbot can be potentially expanded for use in various scenarios. Future development of the Ai-aun chatbot is toward personalized healthcare and remote precision medicine for recognizing and monitoring the health status and behavior of the elderly. The chatbot should allow their family and the authorized caregivers to be in touch with the important updates related to the elderly health.

### Future directions

In future work, the proposed chatbot system should be improved to consider higher-complexity conversations and to cover the requirements of people with more complicated behaviors, lifestyles, and health conditions. Various contexts and environments also affect the chatbot responses. We intend to incorporate the proposed chatbot with the improved models that can provide real-time data by directly monitoring the precise health conditions of individuals and the elderly. The system can be improved by (1) including additional users (family) that can provide additional content and rewards to the elderly person; and (2) establishing cognitive plans that can be evaluated.

### Limitation of the study

The elderly may have benefited during the chatbot use, especially in physical and mental health aspects. These valid chatbots via mobile phone are very easy to learn and use, but the present study’s error tolerance rate, delays and bugs was not measured. This limitation existed but did not have a significant effect on the results. However, it is recommended to address these limitations in future studies.

## Data Availability

Figshare: Chatbot Ai-aun, https://doi.org/10.6084/m9.figshare.19744933.v15 ( Imkome, 2022). This project contains the following underlying data:
- Raw data.xlsx - The training dataset for the chatbot.docx - Demographic data.docx - The results of the interviews.docx - Source Code Aiaun.pdf - AiAunApp - ChitchatAiaun Data are available under the terms of the Creative Commons Attribution 4.0 International license (CC-BY 4.0).
