# Supplementary material for: Validation of a Thai artificial chatmate designed for cheering up the elderly during the COVID-19 pandemic
Source: F1000Res. 2024 Feb 28;11:1411. Originally published 2022 Nov 30. [Version 3] doi: 10.12688/f1000research.127431.3 (PMC11079584; doi:10.12688/f1000research.127431.3)
Supplement: Peer review history

# Reviewer report: Reviewer response for version 3

**DOI:** 10.5256/f1000research.162840.r262292

**Published:** 2024-05-08

**Authors:** Gozde Bacik Yaman

**Affiliations:** 1 Department of Psychiatry, Süleyman Demirel University Faculty of Medicine, Isparta, Turkey

Dear Editor,

 I am grateful for the opportunity to evaluate the study that proposes a mobile chatbot system to support the elderly by encouraging them to access and familiarise themselves with technology to improve their physical and mental health.

 The COVID-19 pandemic has affected physical health and disrupted daily life worldwide by changing individual and social attitudes and behaviors. In such conditions, technological applications are becoming mainstream worldwide to continue social life. Artificial intelligence in healthcare is one of the most popular topics today, and it can provide cost-effective solutions in healthcare. Moreover, chatbot systems offer significant advantages to individuals seeking health information than traditional search engines. They help people understand their health status, improve health-related behaviors, and make informed treatment decisions.

1. The study emphasizes the importance of elderly individuals in Thai culture. It was observed that older people with health problems in Thai culture usually spend most of their time in a private home, which leads to loneliness. Such people depend on other family members and require personal assistance to complete daily tasks and general chores. Therefore, the role of technology is crucial in developing preventive measures against mental health problems in the elderly population.
2. The study used the Post-Study System Usability Questionnaire (PSSUQ) to help users measure their satisfaction with the usability of computer systems and the structure of the preliminary principal factor analysis was confirmed.
3. It was determined in the results section that family members left home for work, and 40.9% of the participants stayed alone at home. After using the Ai-aun chatbot for 15 days, 59.09% of the participants reported being satisfied with the system, which shows that it meets their expectations. In addition, the reasonable response rate of the chatbot was found to be 56.20%. The results revealed that 72.73% of the participants found the chatbot easy to learn and use, and 63.64% thought it was sufficiently autonomous in determining its next course of action. This study shows a moderate usability level of the chatbot.
4. However, the study emphasizes that the elderly may have visual and auditory impairments, which may limit their usage of the chatbot. Also, the fact that the study did not evaluate whether the elderly benefit, especially in terms of mental health, during chatbot use with the results of the stress scale before and after chatbot use can be counted among the limitations.
5. The clinical implications mention that the Ai-aun chatbot can contribute to personalized health services by being developed to recognize and monitor the health status and behaviors of the elderly for future development.

In conclusion, in addition to physical health during the pandemic, it is crucial to improve the mental health of the elderly, who are a more vulnerable group. This study will contribute significantly to the literature on developing care and assistance systems for the elderly in the community during disasters such as the COVID-19 pandemic.

 Gözde Bacık Yaman

Is the work clearly and accurately presented and does it cite the current literature?

Yes

If applicable, is the statistical analysis and its interpretation appropriate?

I cannot comment. A qualified statistician is required.

Are all the source data underlying the results available to ensure full reproducibility?

Yes

Is the study design appropriate and is the work technically sound?

Yes

Are the conclusions drawn adequately supported by the results?

Yes

Are sufficient details of methods and analysis provided to allow replication by others?

Yes

Reviewer Expertise:

Mental Health, mental disorders, stress, percieved stress, behavioural changes in covid 19, videoconference fatigue at covid 19, mental health in the elderly

I confirm that I have read this submission and believe that I have an appropriate level of expertise to confirm that it is of an acceptable scientific standard.

# Reviewer report: Reviewer response for version 2

**DOI:** 10.5256/f1000research.160831.r237283

**Published:** 2024-02-15

**Authors:** Sinan Chen

**Affiliations:** 1 Kobe University, Kobe, Japan

Dear authors, 

 The reviewer provides the following comments:

 (1) In this study, how did the author define the scope of the elderly? Although this article clearly states that the age is over 60 years old. However, is it suitable for older adults with dementia?

 (2) In addition, does the subject live alone? What was their original living situation?

 (3) The reviewer worries that chatbot reminders to take medicine may easily cause medical accidents if the system makes errors. How do you avoid the execution of non-safety projects?

 That is all. Thanks

Is the work clearly and accurately presented and does it cite the current literature?

Partly

If applicable, is the statistical analysis and its interpretation appropriate?

I cannot comment. A qualified statistician is required.

Are all the source data underlying the results available to ensure full reproducibility?

Partly

Is the study design appropriate and is the work technically sound?

Partly

Are the conclusions drawn adequately supported by the results?

Partly

Are sufficient details of methods and analysis provided to allow replication by others?

Yes

Reviewer Expertise:

Smart healthcare, Web application, Human-computer interaction

I confirm that I have read this submission and believe that I have an appropriate level of expertise to confirm that it is of an acceptable scientific standard, however I have significant reservations, as outlined above.

# Response

**Published:** 2024-02-17

**Authors:** Ek-uma Imkome

Dear reviewer,

 Thank you for your thought provoking review. The point-by-point responses are as follows:

 1. The scope of older adults, which we defined on page 5, lines 29-33 of the data collection part as "People aged above 60 years who speak Thai as their first language and can read Thai, was selected. However, the ability to read Thai was a soft requirement because this study focused on communication through the voice interface. Those who were unable to participate in at least 80% of the experiment and had health problems that hindered participation were excluded."

 This chatbot is not provided for older adults with dementia. The subjects of this study were healthy adults in the community, as we state on page 3, line no. 4-6, "Ai-aun can help the elderly adapt to stressful situations during the COVID-19 pandemic by maintaining good mental health, promoting healthy routines, and preventing dementia." We focus on preventing dementia.

 2. The original living situation of the subject was added in the parts of the result as "All samples stay with their family members. In the daytime, the family members leave for work. 40.90% of them live alone and can perform daily activities."

 3. We added the process of maintaining the system to protect the errors. We explained the chatbot safety in terms of the medication response and maintaining the system to protect against errors.

 "For the chatbot system and training, three experts prepared and validated content's Ai-aun's response to the older adults. Afterward, the research teams check the content validity before training the chatbot. The AI-AUN response was programmed and did not create a new sentence for a response while chatting with the older adults as the generative AI. Therefore, there are no errors in the sentence response, especially the medication sentence response. Additionally, maintaining the system to protect the errors was: a) the elderly and the primary caregiver recorded the medication on the application and double-checked for non-high-alert drugs, and high-alert drugs were excluded from the study. The medication data was automatically backed up on the website, b) the research teams asked the primary caregiver to check for medication taken daily, and c) the researcher teams set system checks every week.

 Noteworthy, the chatbot will issue alerts based on the caregiver's time. There are two scenarios in which the reminder system may fail: a) the device is not connected to the Internet: the reminder will not be triggered if the device is not connected to the Internet, and b) the device is not turned on at reminder Time: If the device is not turned on at the scheduled reminder time, the alert will not activate."

 Additionally,  we added "ongoing high-alert drug therapy" to the data collection parts. 

                                          -----------------------------------------------------

# Reviewer report: Reviewer response for version 1

**DOI:** 10.5256/f1000research.139936.r223095

**Published:** 2023-11-24

**Authors:** Sinan Chen

**Affiliations:** 1 Kobe University, Kobe, Japan

The reviewer appreciates the level of detail that the authors provide with accompanying data. Regarding the content described in this article, the reviewer made the following comments:

 (1) Is this conversation robot personalized? For example, how did you obtain pre-training data for emotional word vectors? Additionally, the author should provide a representative history of the conversation.

 (2) Although the author used PSSUQ to evaluate system usability, it is unclear which fine-grained aspects of improving users' mental health conditions are specifically reflected.

 (3) Regarding speech synthesis and recognition technology, what is the error tolerance rate? Will there be delays and bugs? How can the system provide 24-hour virtual care for the elderly who cannot use mobile phones?

 That is all. Thanks

Is the work clearly and accurately presented and does it cite the current literature?

Partly

If applicable, is the statistical analysis and its interpretation appropriate?

I cannot comment. A qualified statistician is required.

Are all the source data underlying the results available to ensure full reproducibility?

Partly

Is the study design appropriate and is the work technically sound?

Partly

Are the conclusions drawn adequately supported by the results?

Partly

Are sufficient details of methods and analysis provided to allow replication by others?

Yes

Reviewer Expertise:

Smart healthcare, Web application, Human-computer interaction

I confirm that I have read this submission and believe that I have an appropriate level of expertise to confirm that it is of an acceptable scientific standard, however I have significant reservations, as outlined above.

# Response

**Published:** 2024-01-07

**Authors:** Ek-uma Imkome

Dear Dr. Sinan Chen,

 We appreciate your time and valuable comments on our manuscript.

 We have carefully reviewed your suggestion. We provide a point-by-point response below:

 (1) Is this conversation robot personalized? For example, how did you obtain pre-training data for emotional word vectors? Additionally, the author should provide a representative history of the conversation.

*Authors: *Thank you for pointing out the critical issue. Per your comment, we added more information on the revised manuscripts on page 6, line no. 19-24.

             “The data pre-training was obtained from the 30 elderly volunteers on the U2T project. The research teams checked for accuracy (general health; 193 datasets; family; 115 datasets; medication; 122 datasets; diet; 137 datasets; exercise; 118 datasets; sleep and rest and other activities; 151 datasets). After obtaining the accurate data set, the researcher teams submitted data to the three experts for the process of content validity testing. The dataset was addressed as the expert comment for the training sessions.”

 (2) Although the author used PSSUQ to evaluate system usability, it is unclear which fine-grained aspects of improving users' mental health conditions are specifically reflected.

*Authors:* Thank you very much for your concern. The mental health improvement state is shown in the research results of another manuscript that is in the process of publication.

 (3) Regarding speech synthesis and recognition technology, what is the error tolerance rate? Will there be delays and bugs? How can the system provide 24-hour virtual care for the elderly who cannot use mobile phones?

*Authors: *Thank you very much. These valuable comments address in limitation of the manuscript as below

 “The elderly may have benefited during the chatbot use, especially in physical and mental health aspects. These valid chatbots via mobile phone are very easy to learn and use, but the present study's error tolerance rate, delays, and bugs was not measured. This limitation existed but did not have a significant effect on the results. However, it is recommended to address these limitations in future studies”.

 That is all. Thanks

# Reviewer report: Reviewer response for version 1

**DOI:** 10.5256/f1000research.139936.r176964

**Published:** 2023-06-19

**Authors:** Sheetal Kusal

**Affiliations:** 1 Symbiosis Institute of Technology, Pune, 412115, India

The paper presents a Thai chatbot for the mental well-being of elderly people. The paper is very interesting to read. Although the authors have tried to make it technically sound, some points must be clarified to make the paper more technically sound.

 Points to be discussed:

1. Some sentences in the paper are repetitive. Could you remove the repetitiveness of sentences?
2. Literature review must be done thoroughly. It is advisable to make an LR table. The authors can refer to the format mentioned in the citation ^1^.
3. In the text pre-processing, the authors have mentioned only segmentation and stop words removal. In NLP, text pre-processing involves many steps to make text appropriate for processing. So, have the authors performed them or which steps? And if not performed, why only stop word removal? Clarify.
4. The authors have mentioned emotion information inserted into the pre-trained word vectors. The authors are expected to elaborate more on how emotion information is inserted into pre-trained word vectors.
5. More discussion is expected on results and performance. 

Is the work clearly and accurately presented and does it cite the current literature?

Partly

If applicable, is the statistical analysis and its interpretation appropriate?

Not applicable

Are all the source data underlying the results available to ensure full reproducibility?

Partly

Is the study design appropriate and is the work technically sound?

Partly

Are the conclusions drawn adequately supported by the results?

Partly

Are sufficient details of methods and analysis provided to allow replication by others?

No

Reviewer Expertise:

Natural language processing, emotional detection, conversational agents, Artificial Intelligence.

I confirm that I have read this submission and believe that I have an appropriate level of expertise to confirm that it is of an acceptable scientific standard, however I have significant reservations, as outlined above.

## References

[1] : AI-Based Conversational Agents: A Scoping Review From Technologies to Future Directions. *IEEE Access* .2022;10: 10.1109/ACCESS.2022.3201144 92337-92356 10.1109/ACCESS.2022.3201144

# Response

**Published:** 2024-01-07

**Authors:** Ek-uma Imkome

Dear Dr. Sheetal Kusal,

 Thank you for your time and valuable feedback on our manuscript. We have carefully reviewed your comments. As suggested, we provide a point-by-point response as below:

 1. Some sentences in the paper are repetitive. Could you remove the repetitiveness of sentences?

*Authors: *Thank you very much. We remove the repetitiveness of sentences on page 6 (line no. 4-6), page 10 (Line no. 4,5,7)

 2. Literature review must be done thoroughly. It is advisable to make an LR table. The authors can refer to the format mentioned in the citation1.

*Authors: *Thank you for your attention. The Literature review is our work and on the process of publication.

 3. In the text pre-processing, the authors have mentioned only segmentation and stop words removal. In NLP, text pre-processing involves many steps to make text appropriate for processing. So, have the authors performed them or which steps? And if not performed, why only stop word removal? Clarify.

*Authors:* Thank you very much. We addressed your comment in the Algorithms and System Design section (Page 6; Line no. 21-22 ) as below:

 “because the sentences were appropriate for content validity by three experts and the research teams before launching the language in NLP. Additionally, we need to check and remove some sentence errors during text pre-processing”.

 4. The authors have mentioned emotion information inserted into the pre-trained word vectors. The authors are expected to elaborate more on how emotion information is inserted into pre-trained word vectors.

*Authors:* Thank you very much. We added the inserted emoji into pre-trained word vectors on page 7 (Line no. 4-10)

 “The visual information in conversational agents may take affective states and thus is an appropriate indication of emojis in texts. The objective is to use emojis in the study for better interpretability of emotional user states, especially for short polysemous phrases of both positive and negative emotions in many scenarios. Understanding willingness, mood, and affect will help conventional agents respond to reliability, validity, and appropriateness. Additionally, Emojis are users’ reported labels of mood and affect to convey emotions in the context of communication interactions”.

 5. More discussion is expected on results and performance.

*Authors: *Thank you very much. We added new references on the part of the discussion to support our results in the text.

 “The agent provides proactive functions, sending messages to help elderly users educate and relax in general health, taking medication, diet, family relationships, exercise, rest, and sleep. Reminiscence strategies are considered challenging when related to design interfaces, conversation logic, and meaningful metrics directed at elderly adults, which benefit from screening and educating them (Kusal et al., 2022; Moreno, Sánchez-Anguix, Alberola, Julián, & Botti, 2024; Otero-González,  Pacheco-Lorenzo, Fernández-Iglesias, & Anido-Rifón, 2024)”.

 References

 1. Kusal S, Patil S, Choudrie J, Kotecha K, et al.: AI-Based Conversational Agents: A Scoping

 Review From Technologies to Future Directions. IEEE Access. 2022; 10: 92337-92356

 2.Otero-González, I., Pacheco-Lorenzo, M. R., Fernández-Iglesias, M. J., & Anido-Rifón, L. E.

 (2024). Conversational agents for depression screening: A systematic review.

 International Journal of Medical Informatics, 181.https://doi.org/10.1016/j.ijmedinf.

 2023.105272

 3. Moreno, J. C., Sánchez-Anguix, V., Alberola, J. M., Julián, V., & Botti, V. (2024). An

 intelligent conversational agent for educating the general public about HIV. Neurocomputing, 563. https://doi.org/10.1016/j.neucom.2023.126902

 ----------------------------------------------
